# Re-thinking care after the pandemic: *a European Care Strategy for Caregivers and Care Receivers*

**DOI:** 10.1007/s12027-023-00744-x

**Published:** 2023-05-30

**Authors:** Eugenia Caracciolo di Torella

**Affiliations:** School of Law, University of Leicester, Leicester, UK

**Keywords:** Long-term care, Childcare, Carers, Gender equality, Covid-19 Pandemic

## Abstract

The importance of care for our sustainability is increasingly discussed by policy makers and academics. For several reasons, however, the law has failed to address it. Accordingly, care has long been in a state of crisis, where the needs of those who require care are not met, and those who care are routinely subject to discrimination and cannot care in a dignified way. The Covid-19 Pandemic has highlighted the extent of the problem. The EU has responded by announcing on 7 September 2022 ‘A European Care Strategy for Caregivers and Care Receivers’. Although not flawless, this initiative is ground-breaking. It is now crucial to sustain momentum and to continue to build on this initiative.

‘If the pandemic taught us one thing, it is that time is precious. And caring for someone you love is the most precious time of all. We will come forward with a new European Care Strategy to support men and women in finding the best care and the best life balance for them.’^1^

## Introduction: the context

Care is an essential and universal feature of our lives. It is essential in the sense that it is ‘an inevitable part’^2^ of life and a ‘basic human need [that] is central to our flourishing.’^3^ More recently, the Commission has referred to it as creating ‘the very ‘fabric that holds our societies together and brings our generations together’.^4^ It is universal because, simply put, it concerns us all:^5^ at some point, during the course of our lives, we all need care to thrive, and in some cases, to survive.^6^

Despite the key importance of care for the sustainability of our lives, the European Union (EU) lacks express competences in this area. Accordingly, the EU legislator has traditionally been unable to address care, either as a concept or in terms of its structure: here there is no legislation and very few guiding principles. In this area, the EU has, at best, provided a forum where Member States could exchange good practices. By contrast, the EU legislator has been able to intervene to regulate the position of carers, where some degree of competence exists, linked to other areas such as employment and gender equality. The approach has been patchy, however. On the one hand, the position of certain carers, namely parents and more specifically mothers, has increasingly been addressed. The last three decades have seen a flurry of legislative measures aimed at supporting them. These include the Directives on Pregnant Workers, Parental Leave and Part Time Workers,^7^ which have been strengthened by a proactive Court of Justice (CJEU).^8^ On the other hand, when it comes to carers of elderly or disabled individuals, namely Long Term Care (LTC), the EU’s engagement has been scarce.^9^ Despite the fact that, already in 1999, they were described as ‘unsung heroes’^10^ and, over the years, a number of policy documents^11^ had emerged, there is little legislation. In other words, measures aimed at carers were organised around a two-tier structure that differentiated between the care of young, healthy children (childcare) and the care of elderly and/or disabled children and dependent adults (LTC) and, as such, it lacked conceptual cohesion.

This position is no longer sustainable, both morally and financially. As a result, care needs are not met and carers cannot provide care in dignified conditions. In other words care is in a state of crisis.^12^ It has taken the COVID-19 Pandemic to highlight the enormity of the problem. The Pandemic has put a magnifying glass on the structural weaknesses of the care systems across Europe. And never was a more compelling case made for the need of robust and resilient formal care services then in those months in 2020-21.

The EU response has been the European Care Strategy for Caregivers and Care Receivers (the Strategy) in order to support men and women in receiving the best care in different life stages.^13^

This article discusses this initiative. It argues that the Strategy is a milestone that shows a welcome change in focus. It is structured as follows. Section 2 starts by explaining what a care strategy should look like and why the EU needs one. To help the reader to put the Care Strategy in context, Section 3 looks at the EU instruments that have paved the way to its adoption and Section 4 focuses on the content of the Strategy. The article concludes that the EU Care Strategy, despite lacking binding force, sends a very powerful message and signals a turning point. However, to ensure its effectiveness more must follow and we cannot afford to lose momentum.

## Should the EU develop a care strategy?

It has become increasingly clear that care can no longer be ignored. Yet, as highlighted in the introduction, the EU does not have the express competence to act in this field: care remains a national issue. Member States are indeed better equipped to address their citizens’ needs, as well as having a better understanding of the available resources. However, it has now become clear that, even in the absence of specific express competences, there are compelling arguments to support the EU’s engagement with care.

First, today care is a global issue that transcends domestic borders. Not only is care rapidly changing into services accessible on the global market,^14^ it is also increasingly being framed within the concept of citizenship rights and welfare state obligations. Second, there is a clear economic argument to support care. Care underpins important EU policies, such as employment and gender equality and the functioning of the internal market. If the EU is serious about ‘closing gender gaps in the labour market’,^15^ it will have to make it possible for *all* individuals to work. At the moment, women continue to experience barriers in the workplace and, unsurprisingly, caring responsibilities are the main reasons for their low market participation.^16^ This is because care continues to be a gendered activity: women are more likely than men to provide more physical, emotional and long-term care.^17^ Third, the Covid-19 Pandemic has reminded us that the very value of care goes beyond its mere economic currency. Any economic argument cannot be ‘decoupled’ from a moral one that values all carers for what they are actually doing, for their contribution to society, rather than focusing on their reduced potential in the employment market.^18^ Care is a form of social capital^19^ that should be embedded in a variety of fields^20^ and should be constructed as, at least, a moral obligation to provide for people who cannot support themselves.^21^ As the EU is no longer merely an economic structure but embraces and promotes human and social rights, the need to address the economic and moral elements of the caring relationship as part of a joined-up discourse becomes more pressing than ever.^22^

Thus, the EU should lead the Member States’ response in this area. This can have a real impact, because the EU can set an agenda, disseminate a policy framework and monitor progress.

## Paving the way towards a Care Strategy

Care has always been identified, and to an extent regulated, as one of the main obstacles to women’s employment. In recent years, however, the realisation that care is essential to the sustainability of our lives, society and economy, and that the law should play a role in promoting it, has gained traction. At EU level, this has prompted the development of a new approach more in tune with the needs of individuals. The European Pillar of Social Right (EPRS)^23^ introduced in 2017 expressly signalled this new approach.^24^ The EPSR contains 20 Principles, covering a wide range of social policy issues. The majority of the EPSR principles have the potential to support and give visibility to care. Amongst these principles, three are particularly relevant to this discussion.^25^ According to Principle 9 (Work Life Balance) ‘Parents and people with caring responsibilities have the right to suitable leave, flexible working arrangements and access to care services. Women and men shall have equal access to special leaves of absence in order to fulfil their caring responsibilities and be encouraged to use them in a balanced way’.

This principle contains two important elements that go beyond the traditional focus on leave provisions.^26^ First, the idea that caregivers need the support of a broad set of social and employment policies, including appropriate long-term care services and flexibility. Second, by referring to carers, rather than simply parents, it acknowledges all types of caring responsibility. Principle 11 (Childcare and Support to Children) is also key. It states that ‘children have the right to affordable early childhood education and care of good quality. Children have the right to protection from poverty. Children from disadvantaged backgrounds have the right to specific measures to enhance equal opportunities’.

Although the EU already had policies that referred to child poverty and to children from disadvantaged backgrounds, the EPSR expressly links them to the concept of work life balance. Finally, Principle 18 states that ‘Everyone has the right to affordable long-term care services of good quality, in particular homecare and community-based services’.

This is perhaps the most innovative because it expressly refers to issues that were traditionally the domain of the Member States. It sends a clear message that high quality, affordable and accessible LTC structures are a right and are an essential pre-requirement to protect individuals’ dignity.

Although the EPSR is not binding, its impact cannot be underestimated. Not only has it contributed to increasing the visibility of care, it has also has broadened the parameters of the discourse in a practical and tangible way. Specifically, it led to the adoption of the Directive on Work Life Balance.^27^ Research has long established that an unsatisfactory work-life balance can have devastating consequences on workers, employers and society at large. It can lead to health problems, poor mental health, absenteeism and poor retention levels. It can also lead to delayed parenting, a decrease in fertility rates and, ultimately, a reduction in labour supply. The Directive, however, is the very first *legislative* instrument that explicitly addresses work-life balance as a self-standing concept. It acknowledges the evolving needs and the diversity of care and carers by modernising the existing framework. It is the first instrument that expressly provides definitions of care and carers. It introduces two flagship rights: rights for carers and paternity leave, and considerably strengthens parental leave and the right to request flexible working arrangements.

The Directive is the first legislative instrument that no longer sees work life balance just as a women’s or, at a stretch, a parents’ problem, but as an issue that affects most workers.

## The European Care Strategy

It was, however, the Covid-19 Pandemic that provoked fresh action in this area. On 15 September 2021, in *The State of The Union* address,^28^ the European Commission president, Ursula von der Leyen, announced the launch of a European Care Strategy. In June 2022, the European Parliament adopted the resolution *Towards a Common European Action on Care*^29^ that urged the Commission and the Member States to put care at the centre of post-pandemic recovery.

It is against this background that the long-awaited European Care Strategy^30^ to ‘support men and women in finding the best balance for them’ was finally adopted by the European Commission on 7 September 2022.^31^ The European Care Strategy is part of the European Pillar of Social Rights’ implementation plan and aims at making the principles on access to good quality and affordable care a reality. It acknowledges that care is the backbone of our society. The Care Strategy departs from the traditional approach used, namely to address issues related to care and caring responsibilities by focusing on the position of *some working carers*. It adopts a holistic approach that, to start with, is reflected in the very name of the strategy which refers to *care* rather than *carers*. This sends the message that the ultimate aim of addressing care is not simply to allow individuals to work, but to develop a system that acknowledges, promotes and protects care and the caring relationship. A holistic approach considers and connects all the different elements of the debate because it acknowledges that to address only some of them, with *ad hoc* interventions, can only produce cosmetic results.

Accordingly, the Care Strategy addresses the needs both of those in need of care, as well as those of carers, because an improvement of the situations of those who provide care, will also benefit those who are cared for. It takes a life-cycle approach that recognises that caring needs and responsibilities do not end with young children, but continue throughout life. In other words, it covers all types of care. Furthermore, it acknowledges the struggle of informal carers. Informal care is still disproportionately undertaken by women, thus contributing to gender inequality. At the same time, it acknowledges that protecting informal carers must go hand in hand with improving and investing in the care sector to ensure that it becomes more resilient and attractive. Finally, a holistic care strategy is underpinned by principles such as dignity, human rights and gender equality both for those who are cared for and their carers. In other words: it leaves nobody behind. It recognises that care is at the very core of the EU’s response to a variety of issues: firstly persistent gender and other inequalities, but also demographic change, the potential of the green and digital transitions, as well as the need to increase resilience to significant external shocks. Thus the Care Strategy is an essential element of the post pandemic recovery that can strengthen and make the Union more resilient.

It is organised in three documents: a Communication on the European Care Strategy and two proposed Recommendations on the access to affordable high-quality long-term care (LTC) and on the Revision of the Barcelona Targets on early childhood education and care, respectively, which have been adopted by the Council on the 8 December 2022. There are, however, a few differences between the proposed and adopted Recommendation.

(i) ***The Communication on the European Care Strategy***

The Communication sets out a vision for the provision of care in Europe. Specifically, it identifies five interlinked areas of actions. First, it sets a strategy for improving care services. In line with a holistic approach, these should be developed and expanded to meet the needs both of young children and those needing long term care in either formal or informal settings. These should be underpinned by the principles of availability, quality, affordability, and accessibility. Whilst these underpinning principles have long been used in this area,^32^ by providing concrete examples, the Communication brings them to life. For instance, when discussing availability, a specific reference is made to the need to provide services in rural and remote areas that might be excluded because of long distances and limited public transport.^33^ Furthermore, for the first time, it is expressly acknowledged that quality must encompass not only infrastructures and services, but also the human interaction between carers and those who are cared for. Therefore, when it comes to early childhood, important elements introduced are inclusivity and the development of non-segregated facilities, where all children can fully benefit. In the case of LTC, the Communication stresses the importance of upholding dignity. Affordability is also presented as crucial for the achievement of a fairer society and to reduce poverty. Finally, the reference to accessibility means enabling those who need care services to actually make use of those that are available. This could be the case of adapting facilities to enable access for people with disabilities.

Second, it acknowledges that improving care services alone is not enough if not accompanied by an improvement in working conditions in the care sector. Care workers are essential to the sustainability of our life, yet their work is underpaid and undervalued. This makes it difficult attracting and retaining staff. Yet, it is estimated that across Europe by 2050, more than 1.6 million of carers would be needed. Thus, it is imperative to improve their working conditions. This can only be achieved if carers are effectively able to exercise their social and employment rights, such as access to better pay and career development opportunities. Strengthening the position of care workers will also positively impact on domestic workers, who are often migrants. However, not all those who provide care are care workers. Informal carers are currently the largest group of care givers in Europe; many try to combine care and work responsibilities, to the detriment of their own physical and mental health. Moreover, the composition of the body of informal carers is very gendered. The extent to which women leave the labour market to undertake unpaid care responsibilities further exacerbates the gender pay gap and the pension gap. This is neither a suitable nor a sustainable solution.

Thus, the third area of action, in line with Principle 9 EPSR, focuses on work life balance as a key component of the care strategy. The Care strategy refers expressly to the Directive on Work Life Balance Directive.

Fourth, the only way to ensure universal access to high quality care, as well as implementing Principles 18 of the European Pillar of Social Rights, is to invest in public care services, which are, for equity and efficiency reasons, best placed to deliver such care. A fundamental revaluing of public care services and recognition that they are an essential public good is needed. Only adequately funded public care services can ensure that people in need of care have autonomy and choice about care provision, whether residential or non-residential care, and that relatives and friends have a choice concerning the care responsibilities they are willing to undertake.

The fifth and final area of action relates to the necessity of having reliable data to monitor progress and develop policies. Data in this area are not, so far, collected routinely and this hinders progress. For example, we understand that care provisions in remote and rural areas are scarce, but more evidence is needed to develop a response to this problem. The Communication emphasises the need to keep reliable and comparable data to increase and monitor progress.

(ii) ***A proposal for a Council recommendation on the Revision of the Barcelona Targets on early childhood education and care***

The Carers Strategy also contains a, now adopted,^34^ proposal for a Recommendation on the Revision of the Barcelona Targets on early childhood education and care (ECEC). These targets, established by the 2002 Barcelona European Council, called for Member States to take into account the demand for childcare facilities and, in line with national patterns of provision, to ‘remove disincentives for female labour force participation and strive, in line with national patterns of provision, to provide childcare by 2010 to at least 90% of children between 3 years old and the mandatory school age and at least 33% of children under 3 years of age’.^35^ The underpinning principle was that early childhood education and care is not only important for children’s cognitive development, but is also key to ensuring women’s participation in the labour market. Indeed, as a result, women’s employment has increased, from 66.1% in 2001 to 67.7% in 2021. However, the gender employment gap remains at a staggering 10.8%.^36^ In 2021, caring responsibilities were responsible for keeping 27.9% of women aged 25-49 outside the employment market, compared to 8% of men. The gender employment gap is matched by the gender care gap, namely the difference in time devoted to unpaid care work by women and men. Furthermore, the implementation of the targets varied significantly across EU countries, with some significantly lagging behind. A revision was therefore necessary.^37^ The (proposed) Recommendation sets new, ambitious, yet realistic, targets. Member States should provide high quality ECEC services in line with national patterns of provisions by 2030. Specifically, they should provide that at least 50% of children below the age of 3 are in ECEC and at least 96% of children between the age of 3 and the starting age for compulsory primary education are in ECEC.^38^

The new targets not only numerically expand upon the previous ones, but also add a further focus to ensure their effectiveness. To start with, the Recommendation includes a specific reference to the intensity of participation in ECEC. Member States are invited to ensure that ECEC services are available for at least 25 hours per week for children below the age of 3, and at least 35 hours per week for children aged 3, to allow parents (women in particular) to participate meaningfully in paid employment. Measures must be affordable, so as not to leave parents out of pocket; accessible to provide a real solution for parents and of good quality, both in terms of staff-child ratio and professionalism of the provider, to increase parents’ confidence in the services. Member States should also strive to close the gap in ECEC attendance between the overall population of children and those children from disadvantaged backgrounds, children at risk of poverty and disabled children or those with special needs, by introducing targeted measures with a view to enabling and increasing participation. Member States should also ensure that ECEC facilities are evenly distributed over the territory across both urban and rural areas. The Communication also refers to the need to provide a comprehensive approach to ECEC where the needs of children of different ages are considered. Thus, Member States should facilitate affordable and high quality out of school care for all primary school children that also includes support with homework. Finally, Member States should ensure that all parents are aware of their rights and entitlements. In order to achieve this, Member States must consider different traditions and backgrounds, as well as abilities in accessing the relevant information, that can impact on parents’ awareness and their decisions.

The Recommendation also acknowledges the need for fair working conditions for the ECEC work force. This is essential to build comprehensive and holistic ECEC measures that can offer a realistic solution to the needs of parents and children.

Whilst the swift adoption of the proposed Recommendation is commendable, there are differences between the two that reflect the difficulties surrounding these issues. First, there are linguistic differences. Whilst in the proposed Recommendation Member States ‘should’ be committed to certain targets and principles, in the adopted Recommendation they are only ‘recommended to’ do so. Secondly, the targets regarding the number of children have been changed, albeit slightly. The Council Recommendation recommended that at least 45% (as opposed to 50% in the proposed Recommendation) of children below the age of three participate in early childhood education and care. Thirdly, the intensity of care has changed: whilst the 25 hours per week for children up to the age of three has remained, the 35 hours for children from the age of three upwards is no longer there. However, support for training programmes for ECEC staff has been expanded.

(iii) ***A proposal for a Council recommendation on the on access to affordable high*-*quality long*-*term care (LTC)***

In line with a holistic approach, the Strategy acknowledges that care does not end with young children. For this purpose, a proposal, now adopted,^39^ for a Recommendation on access to affordable high-quality long-term care (LTC) is included. Although Europe’s rapidly increasing ageing population, meant that a discussion on the need for LTC is not a novelty,^40^ this is the first time that a comprehensive policy has been put forward. Thus, this makes the Recommendation, perhaps, the most innovative element of the Strategy. Indeed, even more than for childcare, LTC systems and standards differ considerably across the Member States. They share, however, similar challenges in relation to their affordability, availability, quality, workforce shortages, organisation and funding. The Covid 19 Pandemic has dramatically emphasised the pressing need to address such challenges. Care deserves support and clear investment. When under-funded and under-staffed, care services are struggling to keep up with the growing demand. As a result, many people are left without options for accessible and affordable care, which increases the risk of old age poverty. Furthermore, with no other care solution, many family members, friends and neighbours, in most cases women, step out of the labour market and take on these responsibilities as informal carers.

Against this background, the proposed Recommendation aims ‘to improve access to affordable, high-quality long-term care’^41^ and is addressed to all people who need it and to all formal and informal carers’^42^ in their efforts to improve accessible, affordable and high-quality long-term care. The Commission recommends that Member States draw up national action plans to improve LTC covering both care structures and carers.

On the one hand, Member States should establish high quality criteria and standards for all LTC settings: these should be timely, comprehensive and affordable in order to allow a decent and dignified standard of living for people with long-term care needs. Member States should also increase the offer and provide a balanced mix of professional long-term care services (homecare, community-based care and residential care). They should also close territorial gaps in the access to long-term care, roll out accessible digital solutions in the provision of care services, and ensure that long-term care services and facilities are accessible to people with disabilities. On the other hand, it aims at improving the situation of carers. This should be achieved by improving working conditions, for example by setting attractive wages, improving initial and continuous education and training, building career pathways through reskilling and upskilling, as well as exploring the possibility of bringing in more care staff, including through legal migration programmes. This would make the LTC profession attractive to both men and women.

Importantly, according to the proposed Recommendation, Member States should ensure a coordination mechanism. Specifically, they should ‘appoint a national long-term care coordinator, supplied with adequate resources and a mandate enabling the effective coordination and monitoring of the implementation of this Recommendation at national level and acting as a contact point at Union level’.^43^ They are also recommended to submit to the Commission a report within 12 months of the adoption of the Recommendation containing a national action plan presenting measures for its implementation.^44^

Also in the case of this proposed Recommendation, there are some differences between the proposal and the adopted Recommendation. As in the case of the Recommendation on Childcare, Member States are only ‘recommended to’ take certain measures, and no longer they ‘should’ do so. The proposal also specified that national coordinators should be appointed to monitor and implement the strategy and that Member States should submit national action plans within 12 months. The approved version, however, refers to national co-ordinators or an ‘appropriate coordination mechanism’ and only recommends Member States to communicate to the Commission the set of measures taken or planned to be implemented, within 18 months.

## Conclusions

There is little doubt that the EU Care Strategy is a milestone that can make a real difference in the way in which the EU engages with care. It identifies care as a value in itself and marks a shift from perceiving care as a problem, to regarding care as a solution that enables individuals to participate, contribute and remain included in society. As such this new approach must be at the heart of the post pandemic recovery.

Perhaps the main drawback of the Care Strategy, is that neither the Recommendation on the revised Barcelona Targets, nor the Recommendation on LTC are binding instruments. Had these instruments been able to create judicially enforceable rights, they would have offered a stronger protection. However, apart from the fact that the EU legislator could have not done so, because of the lack of express competences in this area, rights might not have been the most apt instruments to address an area such as care.

The fact that these instruments lack binding force does not mean that they are empty rhetoric.

These Recommendations can have an ‘indirect legal impact’ on many levels. First, they demonstrate that the issuing institution has made a commitment, thus generating legitimate expectations that objectives must be met. Second, as Sciarra noted nearly 30 years ago, discussing the use of soft law in the context of social and labour law, they ‘can be used as a fulcrum against the standstill of European social policies, when hard law seems to be a faraway achievement.’^45^ Therefore, they can pave the way to further binding action. Arguably, the EU now has a tool to push forward an agenda to design a comprehensive policy and, eventually, a normative framework in this area, rather than merely contribute to care as a by-product of the internal market, as has been the case until now. Finally, the Recommendations provide Member States with an opportunity to rethink and reshape the role and the organisation of care at national level.

Ultimately, the Strategy has unequivocally put care on the EU agenda. This is a welcome step towards building a society where the most vulnerable are not neglected and the pleas of carers are not ignored. But we cannot afford to be complacent and lose momentum.

## Footnotes

1 U. von der Leyen, State of the Union Speech 15.9.2021 available at https://ec.europa.eu/info/strategy/strategic-planning/state-union-addresses/state-union-2021_en (last accessed on 8.5.2023).

2 M. Fineman, *The Autonomy Myth*, New York Press 1994.

3 K. Lynch, ‘Affective Equality: Who Cares?’ (2009) 52 Development, 410.

4 Communication from the Commission on the European Care Strategy COM(2022) 440 final {SWD(2022)} 440 final} available at https://eur-lex.europa.eu/legal-content/EN/TXT/PDF/?uri=CELEX:52022DC0440&from=EN (last accessed on 8.3.2023).

5 See J. Herring, *Caring and the Law* (Hart Publishing, 2013).

6 E. Feder Kittay, *Love’s Labour: Essay on Women, Equality and Dependency* (Routledge, 1999).

7 Council Directive 92/85/EEC O.J. [1992] L348/1, Council Directive 2010/18/EU O.J. [2010] L68/13 repealing Directive 96/34/EC, and Council Directive 97/81/EC O.J. [1998] L14/9 respectively. In addition, policies and soft law measures have been adopted over the years. For a discussion see E. Caracciolo di Torella and A. Masselot, *Caring Responsibilities in European Law and Policy. Who Cares* (Routledge 2020).

8 To name just a few see Case C-104/09 *Roca Álvarez* ECLI:EU:C:2010:561, Case C-221/13, *Mascellani*, EU:C:2014:2286 and C 572/10 *Leone* ECLI:EU:C:2014:117, Case C-363/12 *Z v A Government Department, The Board of Management of a Community School* EU:C:2014:159 and Case C-507/12 *Saint Prix v Secretary of State for Work and Pensions* ECLI:EU:C:2014:2007.

9 R. Horton, ‘Caring for Adults in the EU: Work–Life Balance and Challenges for EU Law’ (2015) 37 *Journal of Social Welfare and Family Law* 356.

10 Case C-160/96 *Molenaar and Fath-Molenaar v Allgemeine Ortskrankenkasse Baden-Württemberg*, Opinion of Advocate General Cosmas, fn 3. ECLI:EU:C:1997:599.

11 For an overview see E. Caracciolo di Torella and A. Masselot, *Caring Responsibilities in European Law and Policy. Who Cares* (Routledge 2020).

12 E. Dowling, *The Care Crisis: What Caused it and How Can we End It?* (London:Verso 2021).

13 A European Care Strategy for Caregivers and Care Receivers, available at https://ec.europa.eu/newsroom/livingdigiworld/items/761492/#:~:text=A%20European%20Care%20Strategy%20for%20caregivers%20and%20care,the%20people%20caring%20for%20them%2C%20professionally%20or%20informally (last accessed on 8.3.2023).

14 See for example, Case C-60/00 *María Martinez Sala* *v Freistaat Bayern* (C-85/96) [1998] ECR-I 2691; *Carpenter v Secretary of State for the Home Department* [2002] ECR I**-**6279, [2003] $2 WLR 267$; Case C-413/99 *Baumbast* *and R v Secretary of State for the Home Department* [2002] ECR I-791; Case C-310/08 *London Borough of Harrow v Ibrahim and Secretary of State for the Home Department* [2010] ECR I-80; Case C-480/08 *Maria Teixeira v London Borough of Lambeth and Secretary of State for the Home Department* [2010] ECR I-83; all these cases involve issues related to care and cross-border situations.

15 Communication from the European Commission, *A Union of Equality: Gender Equality Strategy 2020-2025* COM (2020) 152 final, at 7.

16 European Commission, 2018. Barcelona Objectives on the development of childcare facilities for young children with a view to increase female labour participation, strike a work-life balance for working parents and bring about sustainable and inclusive growth in Europe, available at file:///C:/Users/ecdt6/Downloads/barcelona%20objectives-gp_eudor_WEB_DS0313058ENC_002.pdf (last accessed on 8 March 2023).

17 L. Waddington, ‘Carers, Gender and Employment Discrimination: What does EU Law Offer Europe’s Carers’ in Marie-Ange Moreau (ed), *Before and After the Economic Crisis: What Implications for the ‘European Social Model’?* (Edward Elgar, 2011).

18 G James and E Spruce (2015), ‘Workers with Elderly Dependants: Employment Law’s Response to the Latest Care-giving Conundrum’ 35 *Legal Studies* 463.

19 M. Daly (2000), ‘Caring in the Third Way: the Relationship between Obligation, Responsibility and Care in Third Way Discourse’ 20 *Critical Social Policy* 5.

20 S. Sevenhuijsen (2002), ‘Care as a Good for Social Policy’ 31 *Journal of Social Policy* 251.

21 J Brenner, ‘Democratizing Care’ in J Gornick and M Meyers (eds), *Gender Equality. Transforming Family Divisions of Labour* (Verso, 2009) 177–192, at p 189.

22 A Numhausen-Henning and M Rönnmar (eds), *Normative Patterns and Legal Developments in the Social Dimension of the EU* (Hart Publishing, 2013).

23 Interinstitutional Proclamation on the European Pillar of Social Rights, O.J. [2017] C428/10.

24 See M. Bell (2018), ‘The Principle of Equal Treatment and the European Pillar of Social Rights’, *Giornale di Diritto del Lavoro e delle Relazioni Industriali*, 783 – 810.

25 Amongst other principles, see for example Principle 5 (Secure and Adaptable employment) and Principle 17 (Inclusion of people with disabilities).

26 See for example Article 33 of the EU Charter of Fundamental Rights that simply referred to the leave measures and specifically to maternity and parental leave.

27 Directive (EU) 2019/1158 of the European Parliament and of the Council of 20.6.2019 on work-life balance for parents and carers and repealing Council Directive 2010/18/EU.

28 Available at https://state-of-the-union.ec.europa.eu/system/files/2022-12/soteu_2021_address_en.pdf; see also https://state-of-the-union.ec.europa.eu/state-union-2021_en (last accessed on 8.3.2023).

29 Available at https://www.europarl.europa.eu/doceo/document/A-9-2022-0189_EN.pdf (last accessed on 8.3.2023).

30 Available at https://ec.europa.eu/commission/presscorner/detail/en/ip_22_5169 (last accessed on 8.3.2023).

31 U. von der Leyen, President of European Commission introducing the European Care Strategy, see Communication from the Commission on the European Care Strategy COM (2022) 440 final.

32 See for example the Council Recommendation 92/241/EEC on early childhood education and care OJ L 123, p. 16–18. OJ 2022/C 484/01 available at https://eur-lex.europa.eu/legal-content/EN/TXT/PDF/?uri=CELEX:32022H1220(01)&from=EN (last accessed on 8.3.2023).

33 Communication from the Commission on the European Care Strategy COM(2022) 440 final {SWD(2022)} 440 final} at p. 5.

34 Council Recommendation on early childhood education and care: the Barcelona targets for 2030 2022/C484/01 available at https://eur-lex.europa.eu/legalcontent/EN/TXT/PDF/uri=CELEX:32022H1220(01)

35 Barcelona European Council, available at www.consilium.europa.eu/uedocs/cms_data/docs/pressdata/en/ec/71025.pdf (last accessed on 8.3.2023). However, in 2013 many Countries were still far from reaching these targets; see European Commission, *Barcelona Objectives. The Development of Childcare Facilities for Young Children in Europe with a view to Sustainable and Inclusive Growth*. Report from the Commission to the European Parliament, the Council, the European Economic and Social Committee and the Committee of the Regions (Publications Office of the European Union, 2013), available at: http://ec.europa.eu/justice/gender-equality/files/documents/130531_barcelona_en.pdf (last accessed on 8.3.2023).

36 Eurostat, ‘Employment and Activity by Sex and Age – annual Data’ available at https://data.europa.eu/data/datasets/0c4pfjnlvlu7up3pzeqpa?locale=en (last accessed on 8.3.2023).

37 Such revision had already been announced in the Communication from the Commission *A Union of Equality: Gender Equality Strategy 2020-2025*, COM(2020) 152 final, available at https://ec.europa.eu/commission/presscorner/detail/en/ip_22_5169 (last accessed on 8 March 2023); in the Communication from the Commission, *EU strategy on the rights of the child*, COM(2021) 142 final, available at https://eur-lex.europa.eu/legal-content/EN/ALL/?uri=COM:2021:142:FIN (last accessed on 8.3.2023) as well as the Communication from the Commission, The European Pillar of Social Rights Action Plan COM(2021) 102 final{SWD(2021) 46 final} available at https://eur-lex.europa.eu/resource.html?uri=cellar:b7c08d86-7cd5-11eb-9ac9-01aa75ed71a1.0001.02/DOC_1&format=PDF (last accessed on 8.3.2023).

38 Proposal for a Recommendation on the Revision of the Barcelona Targets on early childhood education and care, at p. 21.

39 Council Recommendation on access to affordable high-quality long-term care 2022/C 476/01 available at https://eur-lex.europa.eu/legal-content/EN/TXT/PDF/?uri=CELEX:32022H1215(01)&from=EN (last accessed on 8.3.2023).

40 E.g. Social Protection Committee and the European Commission, Adequate Social Protection for Long-Term Care Needs in an Aging Society, 18.6.2014, 10406/14 ADD 1; SOC 403 ECOFIN 525.

41 Proposed Recommendation on the on access to affordable high-quality long-term care at 1.

42 Proposed Recommendation on the on access to affordable high-quality long-term care at 2.

43 Proposed Recommendation on the on access to affordable high-quality long-term care at 10(a).

44 Proposed Recommendation on the on access to affordable high-quality long-term care at 11.

45 S. Sciarra (1995), ‘European Social Policy and Labour Law – Challenges and Perspectives’ IV(I) *Collected Courses of the Academy of European Law* 301, 340.

